# Multimodal data integration using machine learning improves risk stratification of high-grade serous ovarian cancer

**DOI:** 10.1038/s43018-022-00388-9

**Published:** 2022-06-28

**Authors:** Kevin M. Boehm, Emily A. Aherne, Lora Ellenson, Ines Nikolovski, Mohammed Alghamdi, Ignacio Vázquez-García, Dmitriy Zamarin, Kara Long Roche, Ying Liu, Druv Patel, Andrew Aukerman, Arfath Pasha, Doori Rose, Pier Selenica, Pamela I. Causa Andrieu, Chris Fong, Marinela Capanu, Jorge S. Reis-Filho, Rami Vanguri, Harini Veeraraghavan, Natalie Gangai, Ramon Sosa, Samantha Leung, Andrew McPherson, JianJiong Gao, MSK MIND Consortium, Yulia Lakhman, Sohrab P. Shah

**Affiliations:** 1 Computational Oncology, Department of Epidemiology and Biostatistics, Memorial Sloan Kettering Cancer Center, New York, NY USA; 2 Weill Cornell/Rockefeller/Sloan Kettering Tri-Institutional MD-PhD Program, New York, NY USA; 3 Department of Radiology, Memorial Sloan Kettering Cancer Center, New York, NY USA; 4 Department of Pathology, Memorial Sloan Kettering Cancer Center, New York, NY USA; 5 Irving Institute for Cancer Dynamics, Columbia University, New York, NY USA; 6 Department of Medical Oncology, Memorial Sloan Kettering Cancer Center, New York, NY USA; 7 Department of Medicine, Weill Cornell Medicine, New York, NY USA; 8 Department of Surgical Oncology, Memorial Sloan Kettering Cancer Center, New York, NY USA; 9 Human Oncology and Pathogenesis Program, Memorial Sloan Kettering Cancer Center, New York, NY USA; 10 Department of Epidemiology and Biostatistics, Memorial Sloan Kettering Cancer Center, New York, NY USA; 11 Department of Medical Physics, Memorial Sloan Kettering Cancer Center, New York, NY USA; 12 Kravis Center for Molecular Oncology, Memorial Sloan Kettering Cancer Center, New York, NY USA

**Keywords:** Machine learning, Cancer, Ovarian cancer, Data integration

## Abstract

Patients with high-grade serous ovarian cancer suffer poor prognosis and variable response to treatment. Known prognostic factors for this disease include homologous recombination deficiency status, age, pathological stage and residual disease status after debulking surgery. Recent work has highlighted important prognostic information captured in computed tomography and histopathological specimens, which can be exploited through machine learning. However, little is known about the capacity of combining features from these disparate sources to improve prediction of treatment response. Here, we assembled a multimodal dataset of 444 patients with primarily late-stage high-grade serous ovarian cancer and discovered quantitative features, such as tumor nuclear size on staining with hematoxylin and eosin and omental texture on contrast-enhanced computed tomography, associated with prognosis. We found that these features contributed complementary prognostic information relative to one another and clinicogenomic features. By fusing histopathological, radiologic and clinicogenomic machine-learning models, we demonstrate a promising path toward improved risk stratification of patients with cancer through multimodal data integration.

## Abstract (web summary)

Shah and colleagues develop a multimodal data integration framework that interprets genomic, digital histopathology, radiomics and clinical data using machine learning to improve diagnosis of patients with high-grade ovarian serous carcinoma.

## Main

High-grade serous ovarian cancer (HGSOC) is the most common cause of death from gynecologic malignancies, with a 5-year survival rate of less than 30% for metastatic disease^1^. Initial clinical management relies on either primary debulking surgery (PDS) or neoadjuvant chemotherapy followed by interval debulking surgery (NACT-IDS). Endogenous mutational processes are an established determinant of clinical course, with improved response of homologous recombination-deficient (HRD) disease to platinum-based chemotherapy and poly-ADP ribose polymerase (PARP) inhibitors^2–4^. More nuanced genomic analyses integrating point mutation and structural variation patterns further refine this stratification into four biologically and prognostically meaningful subtypes^5,6^ including distinct subgroups of HRD, foldback inversion-enriched tumors and those with distinctive accrual of large tandem duplications. Beyond genomic factors, clinical indicators such as patient age, pathological stage and residual disease (RD) status after debulking surgery are also prognostic^7^. However, these clinicogenomic factors alone fail to adequately account for the heterogeneity of clinical outcomes. Identifying patients at risk of poor response to standard treatment remains a critical unmet need. Improved risk stratification models would aid gynecologic oncologists in selecting primary treatment, planning surveillance frequency, making decisions about maintenance therapy and counseling patients about clinical trials of investigative agents.

Beyond clinicogenomic features, multiscale clinical imaging is routinely acquired during the course of care, including contrast-enhanced computed tomography (CE-CT) at the mesoscopic scale and hematoxylin and eosin (H&E)-stained slides at the microscopic scale. Digital forms of these diagnostics present opportunities to develop computational models and test whether integrating these data modalities improves identification of risk groups for HGSOC^8^. At the mesoscopic scale, recent radiologic studies have uncovered quantitative CE-CT features that are predictive of early progression, time to recurrence and overall survival in HGSOC^9–11^. Most studies to date have analyzed the prognostic information captured within adnexal lesions^9,12,13^ or the whole burden of disease^14–16^ and variably use either deep learning or empirically reproducible radiomic features from the Imaging Biomarker Standardization Initiative^17^; however, a radiomic prognostic model based on omental lesions has not yet been developed even though omental implants are ubiquitous in advanced-stage disease. Such a model would be advantageous because it is possible, even for less experienced observers, to delineate omental implants and it would alleviate the need for highly challenging and time-consuming segmentation of the total burden of disease.

At the microscopic scale, H&E-stained tissue biopsies enable pathological diagnosis and are routinely acquired before the start of therapy. A quantitative histopathological study of HGSOC identified patterns of immune infiltration on H&E slides that correlate with mutational subtypes^5^. In other cancer types, studies of whole-slide images (WSIs) have advanced our ability to quantify the histopathological architecture of tumors using deep^18,19^ and interpretable^20,21^ features. Apart from stage, HGSOC lacks independent pre-treatment pathological factors by which to stratify patients^7^ and quantitative approaches thus present an opportunity to systematically develop scaled models that are beyond qualitative human interpretation. Interpretable features are less prone to overfitting in small cohorts and can be more easily interrogated by human pathologists^20,22^.

**Fig. 1:**
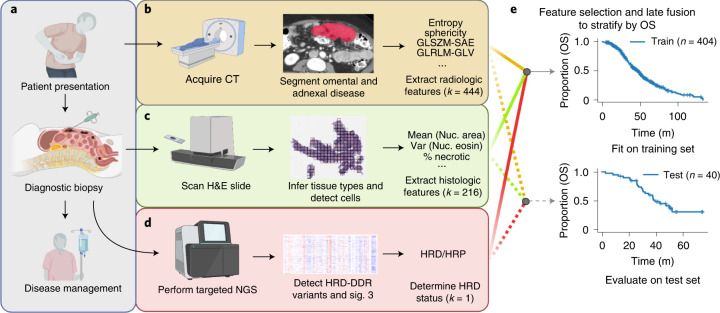
Schematic outline of the study. **a**–**d**, Multiple data modalities were acquired through routine diagnostics to inform clinical decision making (**a**): pre-treatment CE-CT scans of the abdomen and pelvis (**b**), pre-treatment H&E-stained diagnostic biopsies (**c**) and HRD status inferred from hybridization capture-based targeted sequencing or clinical HRD-DDR gene panels (**d**). **e**, Integrated multimodal analyses by late fusion to stratify patients by overall survival. Created with BioRender.com. GLSZM-SAE, gray level size zone matrix small area emphasis; GLRLM-GLV, gray level run length matrix gray level variance; Var, variance; Nuc, nuclear; NGS, next-generation sequencing; LSTs, large-scale state transitions; NtAI, number of subchromosomal regions with allelic imbalance extending to the telomere; LOH, loss of heterozygosity. Source data

Conceptually, genomic sequencing does not account for spatial context and we thus hypothesize that multiscale imaging contains complementary information^8^, rather than merely recapitulating genomic prognostication. We are further motivated by the potential for clinical multimodal machine learning to outperform unimodal systems by combining information from multiple routine data sources. In this work, we set out to study the complementary prognostic information of multimodal features derived from clinical, genomic, histopathological and radiologic data obtained during the routine diagnostic workup of patients with HGSOC (Fig. 1a). We tested the prognostic relevance of ovarian and omental radiomic features derived from CE-CT and developed a model based on omental features (Fig. 1b) and a histopathological model based on pre-treatment tissue samples to risk stratify patients (Fig. 1c). The models were validated on a test cohort and integrated with clinical and genomic information (Fig. 1d) using a late-fusion multimodal statistical framework (Fig. 1e). Our results revealed the empirical advantages of crossmodal integration and demonstrated the ability of multimodal machine-learning models to improve risk stratification of patients with HGSOC.

## Results

### Cohort and clinical characteristics

We analyzed 444 patients with HGSOC, including 296 patients treated at the Memorial Sloan Kettering Cancer Center (MSKCC) and 148 patients from The Cancer Genome Atlas Ovarian Cancer (TCGA-OV) data. The 40 test cases were randomly sampled from the entire pool of patients with all data modalities available for analysis; data from the remaining 404 patients were used for training. The training set contained 160 patients with stage IV disease, 225 with stage III, 10 with stage II, 8 with stage I and 1 with an unknown stage (Supplementary Table 1). The test cohort contained 31 patients with stage IV and 9 with stage III disease^23^. Median age at diagnosis was 63 (interquartile range (IQR) 55–71) years for the training set and 66 (IQR 59–70) years for the test set. In the training cohort, 175 patients received NACT-IDS and the remaining 82 underwent PDS. In the test cohort, 31 received NACT-IDS and 8 underwent PDS. Overall, 61 patients from MSKCC were known to have received PARP inhibitors (Supplementary Table 1). Treatment regimens are not annotated for 148 TCGA patients. Median overall survival (OS) was 38.7 (IQR 25–55) months for training patients and 37.6 (IQR 26–49) months for testing patients. There were 132 training patients and 17 testing patients with censored OS outcomes (Supplementary Table 2).

**Fig. 2:**
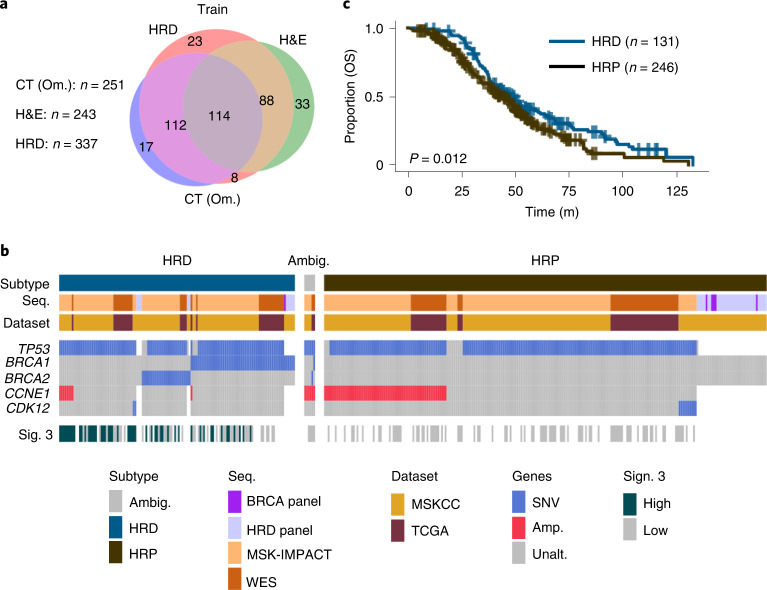
Overview of cohorts and data types acquired. **a**, Venn diagram of patients in the training cohort with available clinical imaging and inferred HRD status. **b**, Inferred subtypes, sequencing modality, dataset of origin, genes with five or more variants and signature 3 status of each patient. Gray represents sequenced genes without the aberrations shown and white represents an unsequenced gene. **c**, Kaplan–Meier analysis on OS stratified by HRD status (*n* = 377 patients). *P* values were calculated using the log-rank test. Sig., mutational signature; SNV, single-nucleotide variation; Amp., copy number amplification; WES, whole-exome sequencing. Source data

**Extended Data Fig. 1:**
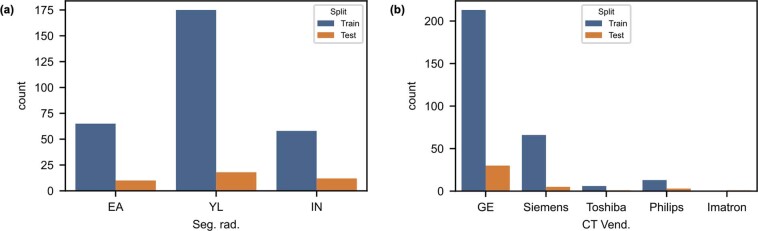
Segmenting radiologist and CT vendor in training and test sets. **a**, Three fellowship-trained radiologists segmented the training (*N* = 298 patients) and test cases (*N* = 40 patients). **b**, Scanner vendors (same number as in preceding panel). Source data

Among 404 patients in the training cohort, 243 patients had H&E WSIs, 245 patients had adnexal lesions on pre-treatment CE-CT and 251 patients had omental implants on pre-treatment CE-CT (Fig. 2a). All 40 patients in the test cohort had omental lesions on CE-CT, H&E WSIs and available sequencing by construction; 29 patients had ovarian lesions on CE-CT. Three gynecologic radiologists volumetrically segmented adnexal lesions and representative omental lesions on all sections containing these lesions (Extended Data Fig. 1a). The training and testing data were acquired with similar CT scanners (Extended Data Fig. 1b).

**Extended Data Fig. 2:**
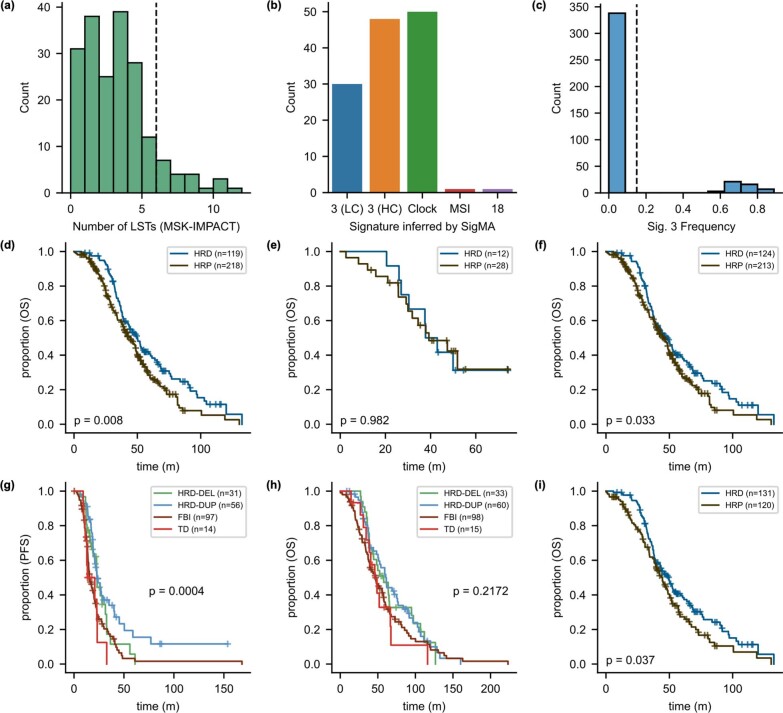
Genomic features of the training and test sets. **a**, Distribution of large-scale state transitions and threshold. **b**, Signature 3 detections by SigMA with high confidence (HC; *N* = 48 patients) and low confidence (LC; *N* = 30 patients), Clock signature (*N* = 50 patients), Signature 18 (*N* = 1 patient), and MSI (*N* = 1 patient). (**c**) Signature 3 frequencies for all TCGA-OV cases with sequencing from^43^ are shown. 338 patients with low Sig. 3 and 47 with high Sig. 3. **d**,**e** Kaplan–Meier analyses of patients by genomic subtype in the training and test sets (*p*-value by log-rank test). **f**, Incorporating thresholded LST counts as indicators of HRD status did not increase the difference in OS of the HRD and HRP curves (*p*-value by log-rank test). **g**, Stratification by PFS using specific mutational subtypes: HRD-Deletion (HRD-DEL), HRD-Duplication (HRD-DUP), Foldback Inversion (FBI), and Tandem Duplications (TD) (*p*-value by multivariate log-rank test). **h**, Stratification by OS using the same mutational subtypes (*p*-value by log-rank test). **i**, Kaplan–Meier analysis by OS for only patients with explicit evidence of HRD or HRP, excluding presumed HRP (*p*-value by multivariate log-rank test). Source data

We used clinical sequencing^24^ to infer HRD status, in particular variants in genes associated with HRD DNA damage response (DDR)^25,26^ such as *BRCA1* and *BRCA2* and those specific to disjoint tandem duplicator- and foldback inversion-enriched mutational subtypes (*CDK12* and *CCNE1* (refs. ^5,27^), respectively; Figs. 1d and 2b,c). We also examined the genomes of 130 patients with appropriate consent for direct evidence of homologous recombination deficiency, namely COSMIC single-base substitution signature 3, which is associated with defective HRD-DDR. In this subset of MSKCC patients, signature 3 was detected by SigMA^28^ with high confidence in 48 cases, detected with low confidence in 30 cases and found not to be the dominant signature in 52 cases (Extended Data Fig. 2b). In the TCGA, signature 3 was high in 6 cases and low in 51 cases (Extended Data Fig. 2c). Patients with available sequencing and without evidence for HRD or homologous recombination proficiency (HRP; *n* = 126) were treated as HRP. Patients with conflicting evidence (*n* = 6) or without sequencing (*n* = 61) were assigned a label of ‘ambiguous’ and excluded from all analyses involving HRD status. In total, the training cohort contained 218 HRP and 119 HRD cases (Fig. 2c). The test set contained 12 HRD and 28 HRP cases. HRD status alone (excluding ambiguous) stratified patients by OS with a c-Index of 0.55 in the training cohort and 0.52 in the test set (without fitting any model parameters; Extended Data Fig. 2d,e). Aberrations specific to distinct endogenous mutational processes also stratified patients as expected: patients with HRP disease had worse outcomes than those with HRD disease (*P* = 7 × 10^−3^; Extended Data Fig. 2g,i).

#### CE-CT imaging feature selection and stratification

**Fig. 3:**
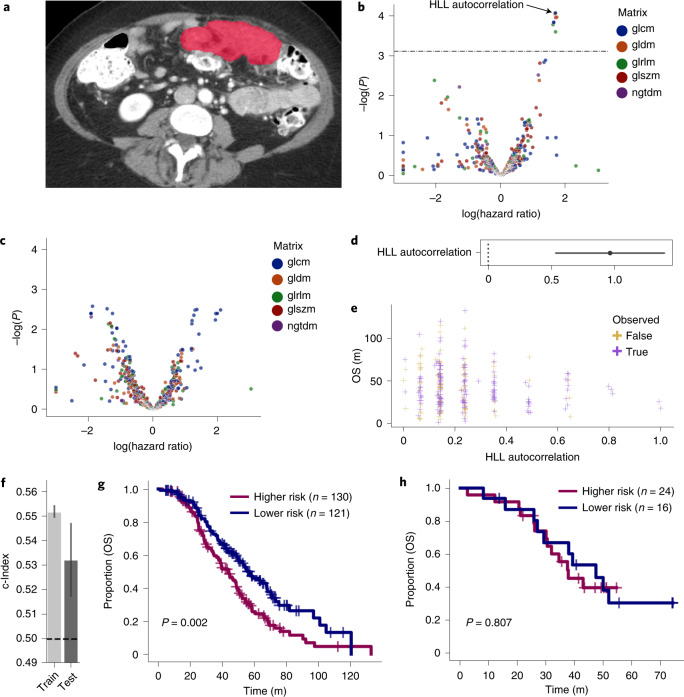
High-autocorrelation omental implants are associated with shorter OS. **a**, Segmented omental lesion (red) on CE-CT. **b**, The log HR is depicted for each radiomic feature derived from omental implants (*n* = 600 features). Features above the line were statistically significant by Cox regression after multiple testing correction of interquartile range-filtered features. **c**, Adnexal radiomic features (*n* = 600 features) were not significant by Cox regression after correction of interquartile range-filtered features. **d**, The hazard ratio with 95% CI as estimated by Cox regression is shown for the feature in the final model, the autocorrelation derived from the gray level co-occurrence matrix for the wavelet-filtered image. **e**, The value of this feature against OS is plotted for patients in the training set (*n* = 251 patients). **f**, Training and test concordance indices for the model are shown; the height of each bar shows the c-Index and the lower and upper points of the respective error bars depict the 95% CI by 100-fold leave-one-out bootstrapping. **g**,**h**, Two risk groups based on the model’s predicted risk score are shown for the training and test sets. *P* values were derived using the log-rank test. glcm, gray level co-occurrence matrix; gldm, gray level dependence matrix; glrlm, gray level run length matrix; glszm, gray level size zone matrix; ngtdm, neighboring gray tone difference matrix. Source data

We began by studying the prognostic relevance of features derived from radiology scans either obtained at our institution (91; 27%) using GE Medical Systems CT scanners or acquired at outside institutions (247; 73%) from a variety of CT scanners (Extended Data Fig. 1 and Supplementary Table 3). The majority of CE-CT scans were acquired with a peak kilovoltage of 120 (median 120 kVp, range: 90–140; Supplementary Table 3) and reconstructed with the standard convolutional kernel using 5-mm slice thickness (median 5 mm; range 2.5–7.5; Supplementary Table 3). Three fellowship-trained radiologists with expertise in gynecologic oncologic imaging manually segmented all adnexal masses and representative omental implants on each pre-treatment CE-CT scan (Figs. 1b and 3a).

**Extended Data Fig. 3:**
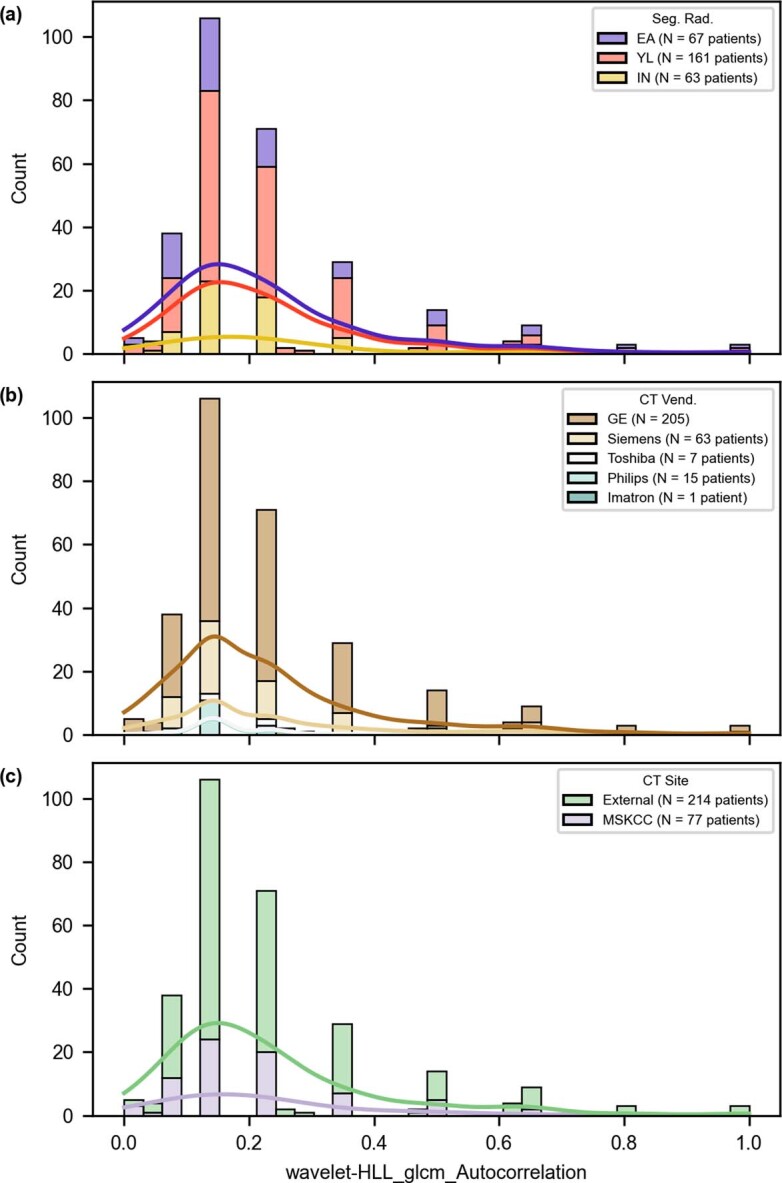
Radiomic feature values by segmenting radiologist, CT scanner, and site. The radiomic feature chosen for the model by **a** segmenting radiologist, **b** CT vendor, and **c** whether the scan was acquired at our institution (MSKCC) or elsewhere. Source data

We extracted radiomic features from Coif wavelet-transformed images, yielding a 444-dimensional radiomic vector per site per patient after filtering by interquartile range. Using the training cohort, we calculated the hazard ratios (HRs) and prognostic significance of omental and ovarian radiomic features using univariate Cox proportional hazards models (Supplementary Table 4)^9^. After correction for multiple hypothesis testing, nine omental features (Fig. 3b) and none of the ovarian features exhibited statistically significant HRs (Fig. 3c). Hence, going forward, we only considered the omental implants. We iteratively fitted and pruned Cox models for multivariable significance on the nine omental features (Algorithm 1), yielding a univariate model based on the autocorrelation of the gray level co-occurrence matrix derived from the high–low–low (HLL) Coif wavelet-transformed^29^ images (Fig. 3d). This feature exhibited a log(HR) of 1.68 (corrected *P* < 0.01; Fig. 3e) and was invariant to CT scanner manufacturers and segmenting radiologists (Extended Data Fig. 3). The model stratified patients in the training and the test sets with concordance indices of 0.55 (95% CI 0.549–0.554) and 0.53 (95% CI 0.517–0.547), respectively (Fig. 3f). Kaplan–Meier analysis of the high- and low-risk groups (as determined by inferred risk) showed statistically different overall survival by the log-rank test (*P* < 0.01) in the training set (Fig. 3g), with median survival of 44 and 57 months, respectively but not in the test set, with median survival of 38 and 47 months, respectively (Fig. 3h).

#### Histopathological tissue-type classifier for interpretable features

**Fig. 4:**
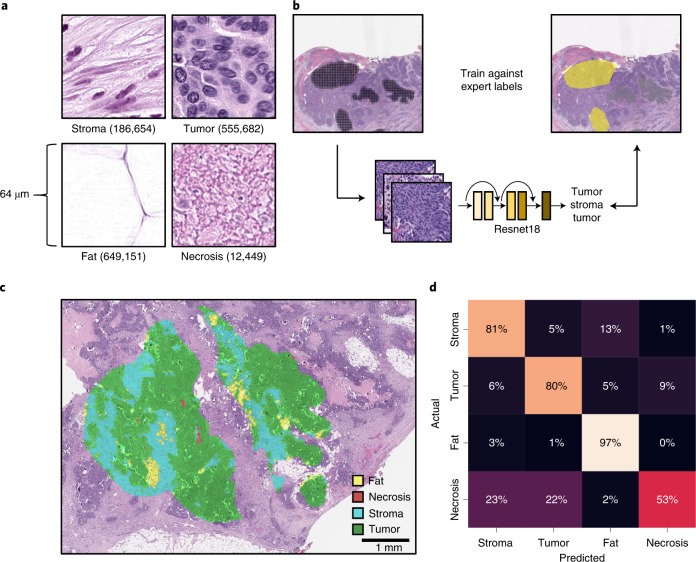
Weakly supervised deep learning accurately infers HGSOC tissue type on H&E. **a**, Annotated tiles normalized using Macenko’s method chosen at random. The number of tiles for each tissue type is shown. **b**, Workflow of ResNet-18 model trained using the annotated regions. **c**, Example of the model’s predictions for an annotated region. **d**, The confusion matrix aggregated across folds of cross-validation for each of the tissue classes. Source data

We next trained a tissue-type classifier from histology images using a weakly supervised approach. We annotated tissue types on 60 H&E WSIs, yielding more than 1.4 million partially overlapping tiles, each measuring 128 × 128 pixels (64 × 64 µm) and containing 4,096 µm^2^ of tissue (Fig. 4a). A ResNet-18 convolutional neural network pretrained on ImageNet (Fig. 4b) classified tissue types with an accuracy of 0.88 (range 0.77–0.95) on pathologist-annotated areas labeled as fat, stroma, necrosis and tumor (Fig. 4c) by fourfold slide-wise cross-validation. Notably, the model correctly identified small regions of fat within stromal annotations and necrotic regions within the tumor, supporting the suitability of weakly supervised deep learning for this task and refining annotations into more granular classifications.

**Extended Data Fig. 4:**
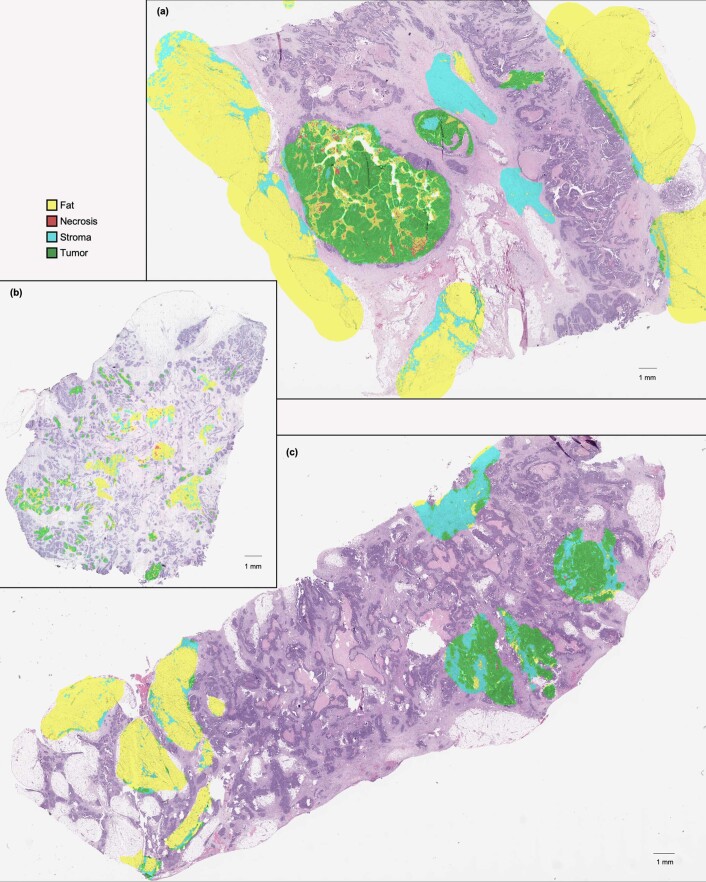
Example cross-validation histopathologic tissue-type classifications. Three samples (a–c) chosen at random from all slides used for cross-validation.

The cross-validation confusion matrix aggregated across folds showed good performance overall (Fig. 4d), with the most significant confusion being necrotic tiles predicted to be tumor and stroma. However, one disadvantage of weakly supervised learning is that neither the training data nor the validation data are exactly labeled. Hence, the cross-validation metrics are not computed against the exact truth. Visual inspection of the predictions were qualitatively concordant with only moderate confusion of necrosis with tumor and stroma (Extended Data Fig. 4).

#### Histopathological stratification

**Fig. 5:**
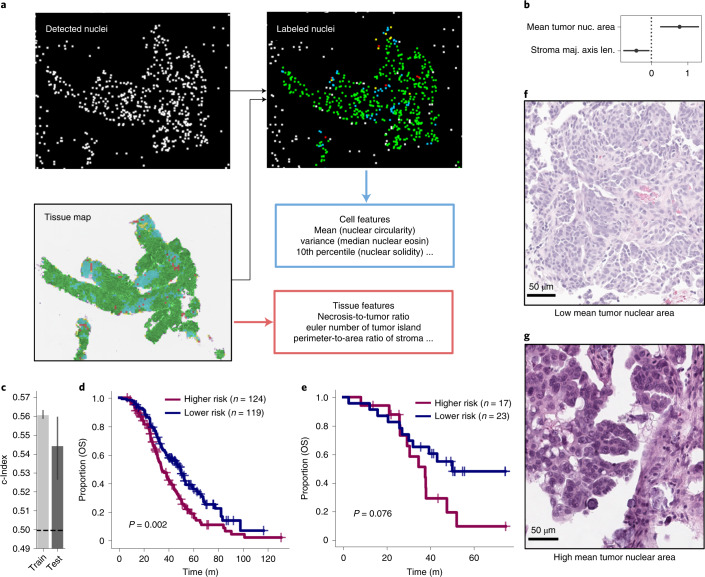
Interpretable histopathological features stratify HGSOC patients by OS. **a**, Tissue map from H&E slides with nuclear detections yielding tissue-type and cell-type features. **b**, Log HRs of the two chosen histological features (with 95% CI as estimated by Cox regression; fit on *n* = 243 patients). **c**, Training and test concordance indices are shown: the height of each bar shows the c-Index and the lower and upper points of the respective error bars depict the 95% CI by 100-fold leave-one-out bootstrapping. **d**,**e**, Kaplan–Meier survival analysis and log-rank test statistics for training (**d**) and test sets (**e**). **f**,**g**, H&E of extreme examples of the model’s inferred mean tumoral nuclear area (scale bar, 50 µm for each image). Source data

**Extended Data Fig. 5:**
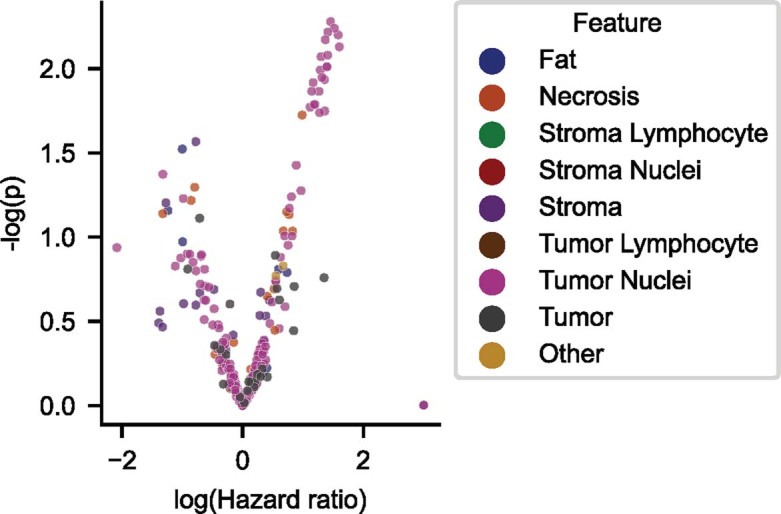
Histopathologic feature discovery. The logarithm of the univariate hazard ratio is depicted for each histopathologic feature (*N* = 281 features) before interquartile range-based filtering, with the cluster in the upper right quadrant comprising primarily features describing tumor nuclear diameter and size. Source data

**Extended Data Fig. 6:**
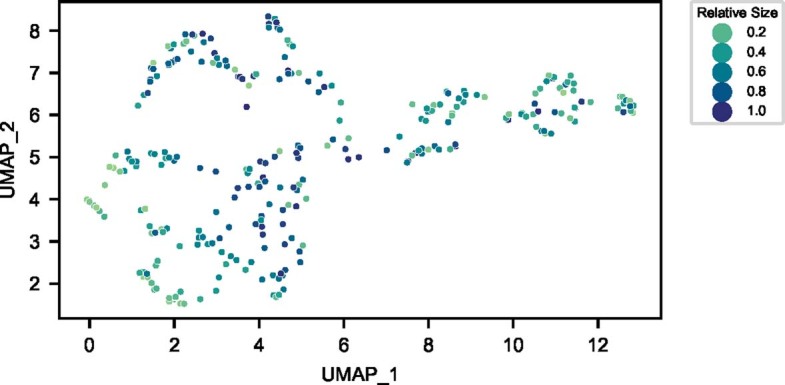
Histopathologic embeddings by specimen size. UMAP embeddings of the two-feature histopathologic signature (for *N* = 283 patients), with each slide’s point colorized by the relative specimen size (here, the quantile of the number of foreground tiles detected). Source data

We applied the tissue-type classifier to the 243 training H&E WSIs of lesions from pre-treatment specimens (Fig. 1c). We combined these inferred tissue-type maps with detected cellular nuclei, yielding labeled nuclei (Fig. 5a). Subsequently, we extracted cell-type features from these nuclei and tissue-type features from the tissue-type maps based on the methods of Diao et al.^20^. This yielded a histopathological vector of 216 features. We next identified the HRs of features using univariate Cox models fitted on slides in the training cohort. Several tissue-type features, such as overall tumoral area, were partially determined by specimen sizes and we thus controlled for this during selection. Of the 24 features with a log(HR) found to be significantly different from 0 with 95% confidence, 20 related to tumor nuclear diameter or size, with larger being associated with shorter OS (Extended Data Fig. 5 and Supplementary Table 5). We again iteratively fitted and pruned Cox models as per Algorithm 1, yielding a multivariable model with two features: the mean tumor nuclear area and the major axis length of the stroma (Fig. 5b). This histopathological signature was not confounded by specimen size (Extended Data Fig. 6). This model stratified the training and test sets, with concordance indices of 0.56 (95% CI 0.559–0.564) and 0.54 (95% CI 0.527–0.560), respectively (Fig. 5c). High- and low-risk groups established based on the inferred risk scores separated well for the training set with a median survival of 34 and 49 months, respectively (Fig. 5d; *P* < 0.01). For the test set, the risk groups trended toward (but did not attain) significantly different separation, with median survival of 37 and 50 months (Fig. 5e; *P* = 0.076). To probe the interpretability of the histopathological features, we investigated the mean tumor nuclear area; we show examples of low (Fig. 5f) and high (Fig. 5g) values, which were associated with better and worse prognosis, respectively.

#### Multimodal prognostication

**Fig. 6:**
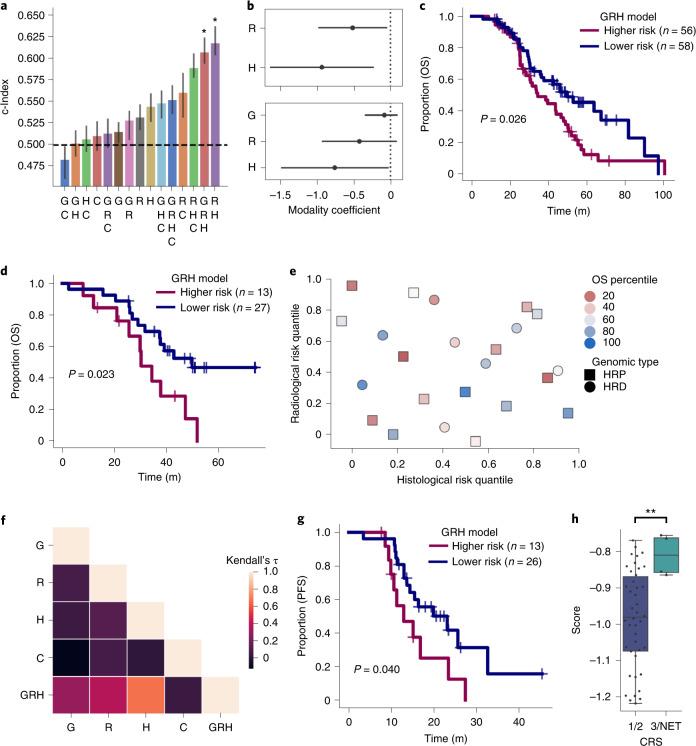
Multimodal integration improves stratification and identifies clinically significant subgroups. **a**, The test c-Indices for integration of combinations of multimodal features is shown: the height of each bar shows the c-Index and the lower and upper points of the respective error bars depict the 95% CI by 100-fold leave-one-out bootstrapping. Asterisks denote 95% confidence of significant ordering of the test set by 1000-fold permutation test. **b**, Log HRs of imaging without (top) and with (bottom) HRD integration. Two modalities are shown fitted on *n* = 122 patients (top) and three are shown fitted on *n* = 114 patients (bottom). **c**, Kaplan–Meier plot comparing high- and low- risk groups determined by the GRH model on the training set. *P* value calculated using the log-rank test. **d**, Kaplan–Meier plot comparing high- and low- risk groups test set. *P* value calculated using a log-rank test. **e**, Unique patients at risk of early death are identified by radiological, histopathological and genomic modalities. Only patients in the test set with uncensored outcomes (*n* = 23 patients) are shown. **f**, Kendall rank correlation coefficient of the risk quantile across pairs of the individual modalities, indicating low mutual ordering information between individual modalities in the training set. **g**, Kaplan–Meier plot of GRH model risk groups on PFS in the test set (one patient has unknown PFS.) *P* value calculated using the log-rank test. **h**, Distributions of GRH model score of low (blue) and high (green) CRS in the training set (*n* = 46 patients). Boxes denote interquartile range, with the center depicting the median and the whiskers denoting the entire distribution excluding any outliers. Significance was assessed by a one-sided Mann–Whitney *U*-test: *P* = 0.0044; **P < 0.01. perm.; permutation test; G, genomic model; H, histopathological model; R, radiological model; C, clinical model; NET, no evidence of tumor. Source data

**Extended Data Fig. 7:**
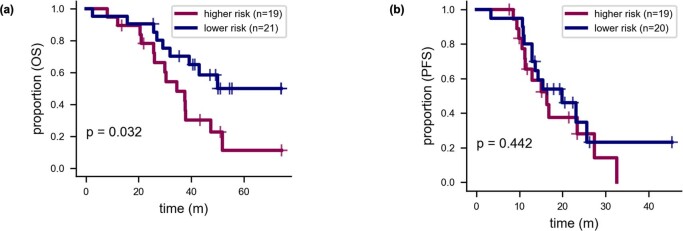
Test performance of histopathologic-radiomic model. **a**, Kaplan–Meier analysis of OS for the RH model’s risk scores (*p*-value by log-rank test). **b**, Kaplan–Meier analysis of PFS for the RH model’s risk scores (*p*-value by log-rank test). Source data

**Extended Data Fig. 8:**
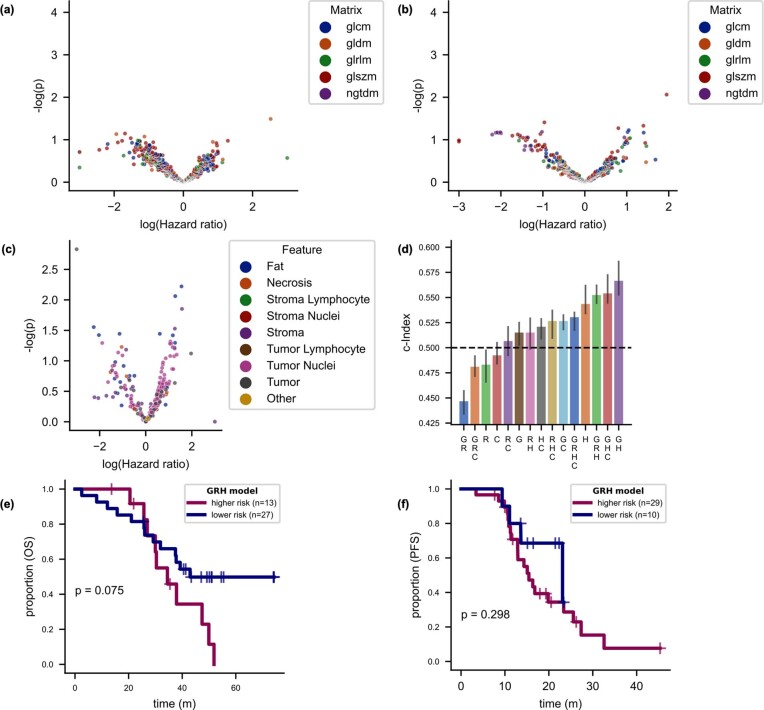
Learning only from cases with full information (*N* = 114) worsens performance. Log hazard ratios for radiomic features derived from **a** omental implants (*N* = 600 features) and **b** adnexal masses (*N* = 600 features; uncorrected *p*-values shown). **c**, Log hazard ratios for histopathologic features (*N* = 281 features; uncorrected *p*-values shown). **d**, Concordance indices for the test set by overall survival. The height of each bar shows the c-Index, and the lower and upper points of the respective error bars depict the 95% C.I. by leave-one-out bootstrapping. Asterisks denote 95% confidence of significant ordering of the test set by 1000-fold permutation test. **e**, KM analysis of OS for the GRH model in the test set. *P*-value calculated using the log-rank test. **f**, KM analysis of PFS for the GRH model in the test set. *P*-value calculated using the log-rank test. Source data

**Extended Data Fig. 9:**
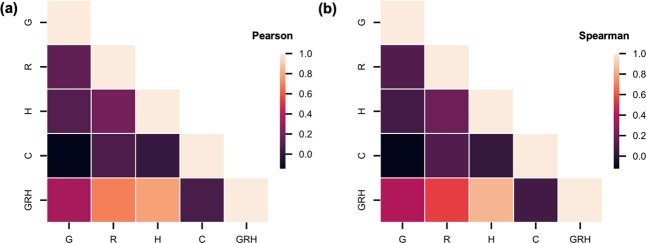
No robust association exists between individual modalities in the training set. **a**, The maximal magnitude of the Pearson correlation between individual modalities is 0.191. **b**, The maximal magnitude of the Spearman correlation between individual modalities is 0.192. Source data

We tested the prognostic significance of patient age, pathological stage, RD status after debulking surgery, NACT-IDS versus PDS treatment paradigm, receipt of PARP inhibitors in the first 2 years after diagnosis and the presence or absence of adnexal lesions (Supplementary Table 6), ultimately training a model on RD status and PARP inhibitor administration. This model stratified the test set with Harrell’s concordance index, *c* = 0.51 (95% CI 0.493–0.528). We then implemented a late-fusion^8^ approach to integrate histopathological, radiomic, genomic and clinical data into multimodal models (Fig. 1e). Specifically, we predicted each patient’s log partial hazard using the Cox model trained using the respective modality, then trained a final Cox model to integrate them (Methods). In the test set, the model combining both imaging modalities (radiomic–histopathological (RH) model) significantly outperformed the HRD status-based model, clinical model and individual imaging models, with a test concordance index of 0.62 (95% CI 0.604–0.638) (Fig. 6a). The model with genomic, radiomic and histopathological (GRH) modalities performed comparably, with a test concordance index of 0.61 (95% CI 0.594–0.625). The histopathological submodel score remained significant upon addition of HRD status (Fig. 6b). The high- and low-risk groups established by the GRH model were significantly different by log-rank test in the training set (median survival of 34 and 50 months, respectively; *P* = 0.026; Fig. 6c). In the test set, the GRH risk groups also showed significantly different OS, with median survival of 30 months for the high-risk group and 50 months for the low-risk group (*P* = 0.023; Fig. 6d). At 36 months, 68% and 34% survived for low- and high-risk groups, respectively, in the test set. The separation of the RH model’s risk groups was inferior (Extended Data Fig. 7). Notably, analysis of only training cases with full information (*n* = 114) resulted in poor performance (Extended Data Fig. 8), reinforcing the ability of late-fusion models to learn in the setting of missing data. No robust association was found between modalities to enable interpolation of missing values (Extended Data Fig. 9).

The c-Indices for individual imaging modalities were similar, but identified distinct patient subgroups with good prognosis (Fig. 6e). This is consistent with radiological and histological features containing complementary information content, whereby some patients with good outcomes were identified as high risk by the radiomic submodel but correctly assigned a lower risk score by the histopathological submodel and vice versa. Patients with HRD and HRP disease were distributed relatively evenly, agnostic to unimodal imaging risk scores.

**Extended Data Fig. 10:**
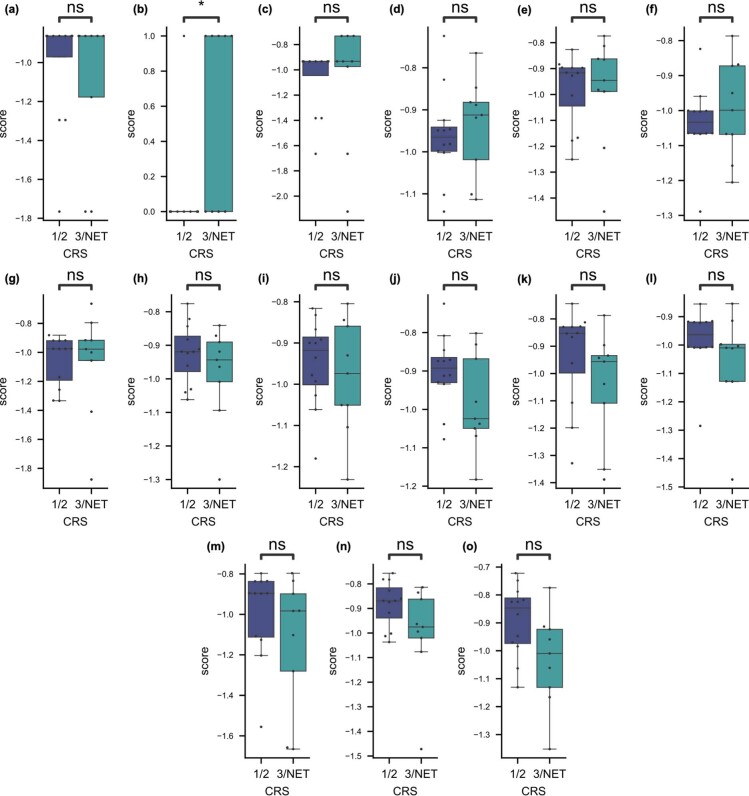
Chemotherapy response scores for all models on the test set. **a**–**o**, for C, G, GC, GH, GHC, GR, GRC, GRH, GRHC, H, HC, R, RC, RH, and RHC models, respectively. The box of each plot depicts the 25th, 50th, and 75th percentiles, and the whiskers depict the entire range except for outlier points beyond 1.5 times the interquartile range past from the median 50% of data. Significance was assessed by a one-sided Mann-Whitney U test without correction for multiple tests. * denotes *p* < 0.05, ** denotes *p* < 0.01, ns denotes *p* > 0.05. *P-*value in **b** is 0.012. Each plot depicts *N* = 9 patients with CRS 3/NET and *N* = 12 patients with CRS 1/2. Source data

Corroborating this, absolute Kendall rank correlation coefficient values were low between individual modalities (<0.14; Fig. 6f), demonstrating that the radiomic and histopathological models ordered patients differently as compared to the genomic model and to one another. The same two risk groups identified by the model in the test set also showed significantly different progression-free survival (PFS) (*P* = 0.040; Fig. 6g). Finally, as an orthogonal validation, the inferred risk of all models except the genomic and genomic–histopathological models associated with pathological chemotherapy response score (CRS) in the training set, including the GRH model (Fig. 6h). The test set had only 21 patients with known CRS and only HRD status exhibited statistically significantly different distributions of CRS by the Mann–Whitney *U*-test in the test set (Extended Data Fig. 10).

## Discussion

Machine learning in cancer prognostics is a growing field with great potential, but the contribution of common diagnostic modalities to multimodal risk stratification remains poorly understood. Here, we show that integrating multiscale clinical imaging and genomic data increases predictive capacity. These results, in addition to the low correlation between risk scores derived from individual modalities, support the hypothesis that clinical imaging contains complementary prognostic information that is independent of clinicogenomic information. Histopathological and radiological imaging characterize the tumor architecture at microscopic and mesoscopic scales, respectively. Therefore, it stands to reason that these data channels complement one another and HRD status, which is derived from spatially agnostic sequencing. The full combined genomic, histopathological, radiological and clinical (GHRC) model did not perform as well as the RH and GRH models, suggesting that multimodality is not a universal guarantee of improved performance^30^. In this case, the most likely reason is that the clinical model (based on history of PARP inhibitor administration and RD status after debulking surgery) does not stratify the test cohort, likely due to its small size. Furthermore, the TCGA cohort did not have these informative clinical variables available. Our late-fusion architecture benefits from few parameters to fit—which reduces overfitting^8^—and the ability to learn from partial information cases, but it cannot gate information from noisy modalities. With larger datasets enabling more parameter fitting without overfitting, mechanisms such as attention can be explored to adaptively adjust unimodal contributions.

In addition to multimodal integration, we presented two unimodal models to stratify patients with late-stage HGSOC using routine clinical imaging, validated these models on a test set and studied the relative contributions of each modality to risk stratifying patients with HGSOC. For radiological imaging, we discovered that omental autocorrelation computed from the gray level co-occurrence matrix derived from the HLL Coif wavelet-filtered image was a prognostic feature. This Imaging Biomarker Standardization Initiative-defined feature^17,31^ has been found to be strongly or very strongly reproducible in multiple studies^32^. It describes the coarseness of the lesion texture and also depends on tissue density. Seven of the other nine omental features with significant log(HR) values were explicitly designed to measure high-density zones and these features did not exhibit log(HR) values significantly different from zero on multivariable regression with the autocorrelation. Hence, the most parsimonious explanation is that higher-density—rather than coarser—omental implants are an adverse prognostic factor, which could be due to more solid tumors with reduced cystic or fatty components. Omental textures captured by autocorrelation may also reflect differing intratumoral heterogeneity.

To our knowledge, previous HGSOC radiomic models have not explored the prognostic information captured within omental implants, relying instead on more demanding segmentations of adnexal lesions or the entire tumor burden. Notably, we found that none of the radiomic features derived from adnexal masses had log(HR) values significantly different from zero after correction for multiple hypothesis testing, which is possibly due to the late stage of this cohort: the omentum is the most common site of metastasis in HGSOC^33^ and may drive further peritoneal seeding. An omental model is advantageous over an adnexal model because omental implants are ubiquitous in advanced-stage disease, even in patients with primary peritoneal high-grade serous cancer that lack adnexal mass(es). Furthermore, an omental implant can be readily segmented even by less experienced observers, whereas adnexal masses can be challenging to distinguish from adjacent loculated ascites, serosal and pouch of Douglas implants and adjacent anatomic structures such as the uterus, especially in the presence of leiomyomas. An omental model is also more practical than a radiomic model based on the whole tumor burden; routine segmentation of the whole tumor volume is impractical in daily practice using current tools due to prohibitively high demand for time and expertise.

For histopathological imaging, we developed an H&E WSI-based model to stratify patients with HGSOC. Although none of the features exhibited log(HR) values significantly different from zero after correction for multiple hypothesis testing, the presence of 20 features highly related to mean tumor nuclear size (such as 60th percentile of tumor nuclear size and 50th percentile of tumor nuclear diameter) with similar HRs in the 24 features with uncorrected significant *P* values for univariate log(HR) values supports the prognostic relevance of tumor nuclear size. This is further supported by the good stratification of the test set. The larger nuclear size may be associated with events such as whole-genome doubling or cellular fusion and warrants direct study of matched genomes and histopathological sections. The major axis length of stroma is difficult to interpret for a two-dimensional slice of tissue but may reflect distinct patterns of disease infiltration into surrounding stroma. We included the trained weights for our HGSOC model and the source code for extension to other cancer types.

This lack of usable large datasets is one of the main challenges for multimodal machine learning in oncology^8^. We have made data from the 296 MSKCC patients with HGSOC available to enable future work toward improving upon the models presented here. Our results demonstrate the benefit of learning from cases with only partial information in multimodal studies: the smaller, full-information subcohort yielded a significantly less-generalizable risk stratification model. Our dataset also offers the advantage of comprising H&E images and CE-CT scans originally acquired at multiple institutions: this improves confidence in the generalizability of the results. Furthermore, we intentionally mined data generated during the standard of care. Using these data instead of specialty research data drastically reduces adoption costs in the clinical workflow for resultant models, but the data were not collected specifically with computational modeling in mind. For example, we included some patients with only germline sequencing of HRD-DDR genes, a clinically relevant but biologically imperfect measure of HRD status: each risk group is enriched for—but not exclusively composed of—the genomic subtype of interest. We expect that clinical whole-genome sequencing will enable more robust genomic analyses.

The improved risk stratification models developed herein show the promise of extracting and integrating quantitative clinical imaging features toward aiding gynecological oncologists in selecting primary treatment, planning surveillance frequency, making decisions about maintenance therapy and counseling patients about clinical trials of investigative agents. The statistical robustness and clinical relevance of the risk groups by both PFS and OS in the test set substantiate the utility of this multimodal machine-learning approach, establishing a proof of principle. Next steps along this line of work include scaled and inter-institutional retrospective cohort assembly for further model training and refinement before prospective validation of clinical benefit in randomized controlled trials^8^.

In summary, we have assembled a multimodal dataset of patients with HGSOC and used this to develop and integrate radiological, histopathological and clinicogenomic models to risk stratify patients. We discovered that the autocorrelation of omental implants on CE-CT and average tumor nuclear size on H&E are prognostic factors, that these modalities are demonstrably orthogonal and that their computational integration improves stratification beyond previously known clinicogenomic factors in a test set. Our results motivate further large-scale studies driven by multimodal machine learning to stratify patients with cancer, both in HGSOC and other cancer subtypes.

## Methods

This study complies with all relevant ethical regulations and its protocols were approved by MSKCC’s Institutional Review Board. Informed consent was waived for this retrospective study and participants were not compensated. Further information on research design is available in the Nature Research Reporting Summary linked to this article.

### MSKCC cohort curation

Patients were eligible for this retrospective study if they had biopsy-proven newly diagnosed HGSOC and at least one of (1) pre-treatment WSIs of H&E depicting high-grade serous carcinoma or (2) pre-treatment contrast-enhanced abdominal/pelvic computed tomography (CE-CT). Most of the MSKCC cohort was sourced from a retrospective clinical database of patients who underwent diagnostic workup and NACT-IDS at our institution. This database also contained information on the RD status after debulking surgery, pathological stage, administration of neoadjuvant chemotherapy and patient age at diagnosis from the electronic medical record. To expand the cohort, we also searched the institutional data warehouse for patients with MSK-IMPACT sequencing and available pre-treatment CT studies or H&E images. In addition to this retrospective curation, 36 patients were also included from the prospective MSK-SPECTRUM project^34^. Pathological stage was unavailable for 14 patients and we instead recorded the clinical stage as recorded in the institutional database for these patients. We also collected the race for all patients from the institutional data warehouse. OS and PFS were calculated using the date of CT as a start date, when available, or the date of pathological diagnosis otherwise.

To collect H&E imaging, we reviewed the electronic health record to find associated pathology cases with peritoneal lesions (primarily omental) and expert pathologists reviewed the slides to select high-quality specimens for digitization. We also reviewed the institutional data repository for scanned slides associated with the diagnostic biopsy and included those containing tumors. All H&E imaging was carried out before treatment.

We subsequently reviewed the associated CE-CT scans for the following the inclusion criteria: (1) intravenous contrast-enhanced images acquired in the portal venous phase, (2) absence of streak artifacts or motion-related image blur obscuring lesion(s) of interest and (3) adequate signal to noise ratio (Supplementary Table 7). All CE-CT imaging was carried out before treatment. All CT scans were available in the digital imaging and communications in medicine (DICOM) format through our institutional picture archiving and communication system (PACS, Centricity, GE Medical Systems v.7.0).

### TCGA cohort selection

From the TCGA-OV project^35^, we selected patients with clinical data annotated in the TCGA Clinical Data Resource^23^, pathological grade 3 and at least one of a diagnostic FFPE H&E WSIs or abdominal/pelvic CE-CT scan in the TCIA^36^. All clinical and demographic information were extracted from the TCGA CDR. Only diagnostic WSIs of formalin-fixed, paraffin-embedded H&E-stained specimens from the TCGA-OV project were included. All H&E imaging was carried out before treatment.

All CT scans met the following the inclusion criteria: (1) intravenous contrast-enhanced images acquired in the portal venous phase, (2) absence of streak artifacts or motion-related image blur obscuring lesion(s) of interest and (3) adequate signal to noise ratio (Supplementary Table 7). All CE-CT imaging was carried out before treatment.

### Inferring HRD status

In the MSKCC cohort, we used MSK-IMPACT clinical sequencing^37^, when available, to infer HRD status. Variant calling for these genes and copy number analysis of *CCNE1* was performed using the standard MSK-IMPACT clinical pipeline (https://github.com/mskcc/Innovation-IMPACT-Pipeline). For patients with appropriate consent for further genomic re-analysis, we also inferred COSMIC SBS3 activity using SigMA (for cases with at least five mutations across all 505 genes)^28^ and searched for large-scale state transitions^38^ using our own pipeline (https://github.com/jrflab/modules/)^39^. We used OncoKB and Hotspot annotations for variant significance^40–42^ in genes involved in HRD-DDR to assign patients to the HRD subtype. Patients with high-confidence dominant signature 3 or at least one significant variant or deep deletion in the HRD-DDR genes^25^ were assigned to the HRD subtype, except when there was evidence that patients belonged to the foldback inversion- or tandem duplicator-enriched subgroups (via *CCNE1* amplification or *CDK12* SNVs, specifically)^5,27^. Patients with conflicting evidence were assigned to the ambiguous subtype and excluded from analysis. Low-confidence signature 3 results were not used for HRD status definition. Incorporating LST thresholding to define HRD status was found to diminish the separation of the HRD and HRP-defined groups in the training set (Extended Data Fig. 2a,f) and thus it was not used in our final HRD status definition. Patients with available results from clinical HRD-DDR panels or *BRCA1/2* sendout panels were assigned HRP unless there were variants of known significance (as determined by the test provider) in at least one reported gene.

In the TCGA cohort, we downloaded copy number alteration (CNA) and SNV data from the TCGA-OV project on cBioPortal for the same set of genes implicated in HRD-DDR^25^, CDK12 and *CCNE1*, again filtering to variants deemed significant by OncoKB. Using these criteria, patients with at least one SNV or deep deletion in HRD-DDR genes were assigned the HRD subtype. Patients without aberrations in these HRD-DDR-associated genes were assigned the HRP subtype. Patients with an SNV in *CDK12* or amplification in *CCNE1* and also with an SNV in at least one of the HRD-DDR genes were assigned the ambiguous subtype and excluded from analysis. Patients without available SNV and CNA data in cBioPortal were assigned to the ambiguous subtype and excluded. We also downloaded COSMIC SBS3 frequencies^43^ from Synapse (syn11801889), which is clearly bimodal (Extended Data Fig. 3c) and patients with SBS3 frequency greater than 15% and without conflicting evidence of HRP were assigned to the HRD subtype.

### Adnexal and omental lesions segmentation

Three fellowship-trained radiologists manually segmented ovarian lesions and representative omental implants on each pre-treatment CE-CT scan for all patients (MSKCC and TCGA-OV/TCIA). Using the Insight Segmentation and Registration Toolkit–SNAP v.3.8.0 software, each radiologist traced the outer contour of ovarian and omental lesions on every tumor-containing axial section. All questions that arose during segmentation were resolved via joint review and consensus.

### Train–test split

Overall, 40 testing cases were sampled randomly before analysis from the patients with available H&E WSI, unambiguous HRD status, known stage and omental lesion on CE-CT. We used this strategy to enable fair comparisons across unimodal and multimodal models, preventing spurious differences in test concordance indices due to patient exclusion for some models but not for others. We included both TCGA-OV and MSKCC cases in the training and test sets; this is because only four TCGA cases had complete information from all modalities and thus could not support a fully external test set.

### Radiological feature extraction

We converted all DICOM series to volumetric images in Hounsfield Units (HU) and applied an abdominal window (level 50 and width 400). Using PyRadiomics^44^, we resampled images to isotropic 1-mm^3^ voxels using the Simple ITK B-spline interpolator and binned images with bin size of 25 HU. We extracted features in three-dimensions from Coif wavelet-transformed images. We extracted features from the gray level size zone^45^, neighboring gray tone difference^46^, gray level run length^47^, gray level dependence^48^ and gray level co-occurrence^49^ matrices, yielding a representation of each study’s representative omental lesion(s) or individual adnexal lesion(s).

### Histopathological annotation

Two expert pathologists partially annotated 60 H&E WSIs using the MSK Slide Viewer^50^. The approach was to label example regions of necrosis, lymphocyte-rich tumor, lymphocyte-poor tumor, lymphocyte-rich stroma, lymphocyte-poor stroma, veins, arteries and fat with reasonable but imperfect accuracy. We exported these annotations as bitmaps and converted them to GeoJSON objects. We amalgamated lymphocyte-rich/poor tumor labels and lymphocyte-rich/poor stroma labels for training and omitted vessels from the training data for the models presented in this work. We next used these annotations to generate tissue-type tiles.

### Training the histopathological tissue-type classifier

We generated tiles measuring 64 µm × 64 µm (128 × 128 pixels) with 50% overlap, using the above annotations to delineate regions to be tiled. No other tile sizes were explored; this size was chosen because it offered good resolution while still depicting multiple cells in each tile. Putative tile squares within an annotation but with <20% foreground as assessed by Otsu’s method were not tiled. Macenko stain normalization was used. We trained a ResNet-18 model (pretrained on ImageNet) for 30 epochs with a learning rate of 5 × 10^−4^, 1 × 10^−4^ L2 regularization and the Adam optimizer. The objective function was class-balanced cross entropy and we used mini batches of 96 tiles on a single NVIDIA Tesla V100 GPU. We used fourfold, slide-wise cross-validation for model evaluation and hyperparameter tuning. We selected the number of epochs to train the final model using the epoch with the highest lower 95% CI bound estimated using the mean and s.d. of the cross-validation F1 scores. We trained the model on tiles from all 60 slides for 21 epochs.

### Histopathological feature extraction and selection

We tiled the WSIs associated with the patients in this cohort without overlap, performing inference using mini batches of 800 across four NVIDIA Tesla V100 GPUs. We used Macenko stain normalization for all slides because staining intensity differences from our predominantly MSKCC-based training cohort confounded inference. We assembled tile predictions into downscaled bitmaps, which were then used to calculate tissue-type features in an approach based on previous work^20^. We included the region properties from scikit-image^51^ for both the largest connected component and the entirety of each tissue type. We also calculated features such as the area ratio of one tissue type to another and the entropy of tumor and stroma. Using the StarDist method^52^ for QuPath^53^, we segmented and characterized individual nuclei, using nuclei with a detection probability greater than 0.5. We used a lymphocyte classifier trained iteratively using manual annotations to distinguish lymphocytes from other cells. We assigned a tissue parent type to each nucleus using the inferred tissue-type maps and calculated aggregative statistics by tissue type and cell type of the QuPath-extracted nuclear morphological and staining features, such as variance in eosin staining or circularity. Together, these cell type features and tissue-type features based on tumor, stroma and necrosis constituted the histopathological embedding for each slide.

### Clinical data encoding

RD status after debulking surgery was encoded as a binary variable, where patients with ≤1 cm RD (including complete gross resection) were assigned a value of 1 and patients with >1 cm RD were assigned a value of 0. The presence of adnexal lesions on CE-CT was also included as a binary variable. Age at diagnosis was modeled as a continuous variable scaled by the training set range. Tumor stage was encoded as one-hot categorical variables for I, II, III, IV and unknown. Similarly, the primary treatment approach was encoded as a one-hot categorical variable with values NACT-IDS, PDS and unknown.

### Feature selection

The same strategy was used to select radiomic, histopathological and clinical features. For each feature, we fitted a univariate Cox proportional hazards model to the full training set using the Python Lifelines package without regularization and we plotted the univariate coefficient and significance confidence. For features whose model failed to converge, we re-attempted fitting with L2 regularization *c* = 0.2 and any model still failing to converge was assigned a log(HR) of 0 and *P* value of 1. For histopathology, we controlled for relative specimen size by including it in each Cox model. We next removed features with scaled IQR below 0.1. Subsequently, for radiomics, which is the largest feature space, we used the Benjamini–Hochberg method to correct for multiple hypothesis testing^54^. Taking the ordered list of features significant with 95% confidence, we next applied Algorithm 1 to select features, yielding modality signatures with low multicollinearity.

#### Algorithm 1. Multivariable model selection procedure

**Input:** A list of unique candidate features ordered by *P* value *f*_*i*_ where *i* ∈ [1*,k*].

**Output:** A list of features significant with confidence *α* on multivariable regression *g*_*j*_ where *j* ∈ [1*,l*] and *l*≤*k*.

**Require:** *k*≥1 *i*←1 *j*←1

  **while** *i*≤*k* **do**

  *g*_*j*_←*f*_*i*_

   ***P***←significance(**g**) ▷ significance assessed by Cox regression

  **if** *P*_*j*_<*α* **then** *j*←*j*+1

  **end if**

  *i*←*i*+1

 **end while**

The only modification to this procedure occurred for the ablation experiment to test the importance of learning from the partial information cases: we used a threshold of 0.31 for clinical features as none was significant with *P* < 0.05 and we did not correct for multiple hypothesis testing in the omental radiomic features during the ablation experiment as none would be significant by this metric.

### Survival modeling

We used linear Cox proportional hazards models with L2 regularization (*c* = 0.5) and no L1 regularization for all multimodal and unimodal models. No submodel was fitted for the genomic modality; patients assigned to the HRP subtype were designated high risk (risk score 1.0) and patients assigned to the HRD subtype were designated low risk (risk score 0). No interaction terms were used.

We used Kaplan–Meier analysis to determine whether each model stratified patients into clinically significant groups. To delineate group membership, we tested percentile thresholds in {0.33, 0.34, …, 0.64, 0.65 0.66}, choosing the value that maximized significance of the separation in the training set by the log-rank test. This was performed individually for OS and PFS, where relevant. *P* values for concordance indices were calculated using 1000-fold permutation tests. The 95% CI for c-Indices were calculated using 100-fold leave-one-out bootstrapping. All *P* values for Kaplan–Meier analysis were calculated by the multivariate log-rank test. *P* values for covariate significance in Cox proportional hazards models are reported for models fitted with *c* = 0.5. The fraction surviving was estimated using linear interpolation.

### Multimodal integration

We chose a late-fusion approach to increase unimodal sample sizes available for parameter estimation^8^. Parameters for unimodal submodels were estimated using all available unimodal data (radiomic parameters were estimated across the 251 training CT cases with omental lesions and histopathological parameters were estimated across the 243 training H&E cases), where each submodel inferred a partial hazard for each patient. The negative partial hazard was used to enable compatibility with the concordance index as implemented in the lifelines Python package^55^. For the second-stage late-fusion model, we estimated parameters for a multivariate Cox model integrating the negative log partial hazards inferred by each modality using only the intersection set of patients.

### Statistics and reproducibility

No statistical method was used to predetermine sample size. Data were excluded from the analyses only for the reasons detailed above and before any machine-learning modeling. The training and test sets were chosen at random from the patients with all four data modalities available. The investigators were not blinded to allocation during outcome assessment. Data distributions were not assumed to be normal for any tests. The hazards were assumed to be proportional for survival modeling, but this was not formally tested.

Analysis was conducted in QuPath v.0.2.3 (with the StarDist extension), ITK SNAP v.3.8.0 and custom code written in Python v.3.9.4 (using Pandas v.1.2.4, NumPy v.1.20.2, PyTorch v.1.5.1, TorchVision v.0.6, OpenSlide v.1.1.1, Seaborn v.0.11.1, Matplotlib v.3.4.2, SciPy v.1.6.3, scikit-learn v.0.24.0, PyRadiomics v.3.0 and Lifelines v.0.25.7).

### Reporting summary

Further information on research design is available in the Nature Research Reporting Summary linked to this article.

## Supplementary information

Supplementary Information List of MSK MIND Consortium members.

Reporting Summary

Supplementary Tables. Clinical and demographic characteristics of cohorts (tab 1). Continuous values are described by the median. Percentage values may not sum to 100% due to rounding error. Outcomes of cohorts (tab 2). Censored durations are also included in survival descriptions. CT scanner parameters (tab 3). Continuous values are summarized by median (IQR) (min−max). Log-rank statistics for omental radiomic features (tab 4). Log-rank statistics for histopathologic features (tab 5). Log-rank statistics for clinical features (tab 6). CT scan exclusion criteria (tab 7). Of 445 cases reviewed, 107 cases were excluded, leaving 338 for analysis.

## Data Availability

DNA sequencing, H&E WSI and CT data that support the findings of this study have been deposited at Synapse (Sage Bionetworks) under accession code syn25946117. Additional H&E WSI, CT imaging and genomic data were derived from the TCGA Research Network: http://cancergenome.nih.gov/ and The Cancer Imaging Archive: https://www.cancerimagingarchive.net/. Raw data from MSK-IMPACT performed in the CLIA laboratory in the Department of Pathology is not currently permitted in public repositories because ethical and legal implications are still being discussed at an institutional level; thus, the derivative features related to HRD status are shared in the repository. Source data have been provided as Source Data files. All other data supporting the findings of this study are available from the corresponding author on reasonable request. Source data are provided with this paper.

## Source data

Source Data Fig. 1 OS outcomes.
Source Data Fig. 2 Modality availability, OncoPrint and genomic subtype versus OS.
Source Data Fig. 3 Radiomic feature log HRs, radiomic model forest plot and radiomic score versus OS and c-Indices.
Source Data Fig. 4 Tissue-type confusion matrix.
Source Data Fig. 5 Histopathological model forest plot and histopathological score versus OS and c-Indices.
Source Data Fig. 6 Multimodal c-Indices, forest plots, multimodal risk score versus OS/PFS, unimodal score comparison and CRS versus multimodal score and Kendall’s tau correlation.
Source Data Extended Data Fig. 1 Segmenting radiologist and scanner vendor.
Source Data Extended Data Fig. 2 Extended genomic analyses.
Source Data Extended Data Fig. 3 Radiomic score by vendor, segmenting radiologist and acquisition site.
Source Data Extended Data Fig. 5 Uncorrected histopathological feature log HRs.
Source Data Extended Data Fig. 6 UMAP embeddings, histopathological features and relative slide size.
Source Data Extended Data Fig. 7 RH model Kaplan–Meier analyses for test set by OS and PFS.
Source Data Extended Data Fig. 8 Feature log HRs, c-Indices and OS/PFS by GRH model trained on only subset with full information.
Source Data Extended Data Fig. 9 Pairwise Pearson and Spearman correlations between modalities.
Source Data Extended Data Fig. 10 CRS categories versus individual model scores in the test set.

## Code availability

All code necessary for the analyses is shared without access restrictions in a public repository (https://github.com/kmboehm/onco-fusion).

## References

[1] National Cancer Institute. *Cancer Stat Facts*. https://seer.cancer.gov/statfacts/

[2] Moore K (2018). Maintenance olaparib in patients with newly diagnosed advanced ovarian cancer. N. Engl. J. Med..

[3] Gallagher DJ (2011). Survival in epithelial ovarian cancer: a multivariate analysis incorporating BRCA mutation status and platinum sensitivity. Ann. Oncol..

[4] Gorodnova TV (2015). High response rates to neoadjuvant platinum-based therapy in ovarian cancer patients carrying germ-line BRCA mutation. Cancer Lett..

[5] Zhang AW (2018). Interfaces of malignant and immunologic clonal dynamics in ovarian cancer. Cell.

[6] Macintyre G (2018). Copy number signatures and mutational processes in ovarian carcinoma. Nat. Genet..

[7] Kobayashi Y, Banno K, Aoki D (2021). Current status and future directions of ovarian cancer prognostic models. J. Gynecol. Oncol..

[8] Boehm, K. M., Khosravi, P., Vanguri, R., Gao, J. & Shah, S. P. Harnessing multimodal data integration to advance precision oncology. *Nat. Rev. Cancer*10.1038/s41568-021-00408-3 (2021). 10.1038/s41568-021-00408-3 PMC8810682 34663944

[9] Lu H (2019). A mathematical-descriptor of tumor-mesoscopic-structure from computed-tomography images annotates prognostic- and molecular-phenotypes of epithelial ovarian cancer. Nat. Commun..

[10] Rizzo, S. et al. Computed tomography based radiomics as a predictor of survival in ovarian cancer patients: a systematic review. *Cancers*10.3390/cancers13030573 (2021). 10.3390/cancers13030573 PMC7867247 33540655

[11] Rizzo S (2018). Radiomics of high-grade serous ovarian cancer: association between quantitative CT features, residual tumour and disease progression within 12 months. Eur. Radiol..

[12] Wei W (2019). A computed tomography-based radiomic prognostic marker of advanced high-grade serous ovarian cancer recurrence: a multicenter study. Front. Oncol..

[13] Wang S (2019). Deep learning provides a new computed tomography-based prognostic biomarker for recurrence prediction in high-grade serous ovarian cancer. Radiother. Oncol..

[14] Vargas HA (2017). A novel representation of inter-site tumour heterogeneity from pre-treatment computed tomography textures classifies ovarian cancers by clinical outcome. Eur. Radiol..

[15] Meier A (2019). Association between CT-texture-derived tumor heterogeneity, outcomes, and BRCA mutation status in patients with high-grade serous ovarian cancer. Abdom. Radiol..

[16] Zargari A (2018). Prediction of chemotherapy response in ovarian cancer patients using a new clustered quantitative image marker. Phys. Med. Biol..

[17] Zwanenburg A (2020). The image biomarker standardization initiative: standardized quantitative radiomics for high-throughput image-based phenotyping. Radiology.

[18] Fu Y (2020). Pan-cancer computational histopathology reveals mutations, tumor composition and prognosis. Nat. Cancer.

[19] Courtiol P (2019). Deep learning-based classification of mesothelioma improves prediction of patient outcome. Nat. Med..

[20] Diao JA (2021). Human-interpretable image features derived from densely mapped cancer pathology slides predict diverse molecular phenotypes. Nat. Commun..

[21] Sammut, S.-J. et al. Multi-omic machine learning predictor of breast cancer therapy response. *Nature*10.1038/s41586-021-04278-5 (2021). 10.1038/s41586-021-04278-5 PMC8791834 34875674

[22] Rudin C (2019). Stop explaining black box machine learning models for high stakes decisions and use interpretable models instead. Nat. Mach. Intel..

[23] Liu J (2018). An integrated TCGA pan-cancer clinical data resource to drive high-quality survival outcome analytics. Cell.

[24] Zehir A (2017). Mutational landscape of metastatic cancer revealed from prospective clinical sequencing of 10,000 patients. Nat. Med..

[25] Heeke, A. L. et al. Prevalence of homologous recombination–related gene mutations across multiple cancer types. *JCO Precis. Oncol.*10.1200/PO.17.00286 (2018). 10.1200/PO.17.00286 PMC6139373 30234181

[26] Riaz N (2017). Pan-cancer analysis of bi-allelic alterations in homologous recombination DNA repair genes. Nat. Commun..

[27] Funnell, T. et al. Integrated structural variation and point mutation signatures in cancer genomes using correlated topic models. *PLoS Comput. Biol*. 10.1371/journal.pcbi.1006799 (2019). 10.1371/journal.pcbi.1006799 PMC6402697 30794536

[28] Gulhan DC, Lee JJ-K, Melloni GEM, Cortés-Ciriano I, Park PJ (2019). Detecting the mutational signature of homologous recombination deficiency in clinical samples. Nat. Genet..

[29] Beylkin G, Coifman R, Rokhlin V (1991). Fast wavelet transforms and numerical algorithms I. Commun. Pure Appl. Math..

[30] Wang, W., Tran, D. & Feiszli, M. What makes training multi-modal classification networks hard? In *2020 IEEE/CVF Conference on Computer Vision and Pattern Recognition (CVPR)* (IEEE, 2020).

[31] Soh L-K, Tsatsoulis C (1999). Texture analysis of SAR sea ice imagery using gray level co-occurrence matrices. IEEE Trans. Geosci. Remote Sens..

[32] The image biomarker standardisation initiative (IBSI) 0.0.1dev documentation. https://ibsi.readthedocs.io/ (2021).

[33] Bowtell DD (2015). Rethinking ovarian cancer II: reducing mortality from high-grade serous ovarian cancer. Nat. Rev. Cancer.

[34] Vázquez-García, I. et al. Immune and malignant cell phenotypes of ovarian cancer are determined by distinct mutational processes. Preprint at *bioRxiv*10.1101/2021.08.24.454519 (2021).

[35] Cancer Genome Atlas Research Network. (2011). Integrated genomic analyses of ovarian carcinoma. Nature.

[36] Prior, F. W. et al. TCIA: an information resource to enable open science. In *35th Annual International Conference of the IEEE Engineering in Medicine and Biology Society (EMBC)* 1282–1285 (IEEE, 2013). 10.1109/EMBC.2013.6609742 PMC4257783 24109929

[37] Cheng DT (2015). Memorial Sloan Kettering-integrated mutation profiling of actionable cancer targets (MSK-IMPACT): a hybridization capture-based next-generation sequencing clinical assay for solid tumor molecular oncology. J. Mol. Diagn..

[38] Popova T (2012). Ploidy and large-scale genomic instability consistently identify basal-like breast carcinomas with BRCA1/2 inactivation. Cancer Res..

[39] Mandelker D (2019). The landscape of somatic genetic alterations in breast cancers from CHEK2 germline mutation carriers. JNCI Cancer Spectr.

[40] Chakravarty, D. et al. OncoKB: a precision oncology knowledge base. *JCO Precis. Oncol.*10.1200/PO.17.00011 (2017). 10.1200/PO.17.00011 PMC5586540 28890946

[41] Chang MT (2018). Accelerating discovery of functional mutant alleles in cancer. Cancer Discov..

[42] Chang MT (2016). Identifying recurrent mutations in cancer reveals widespread lineage diversity and mutational specificity. Nat. Biotechnol..

[43] Alexandrov LB (2020). The repertoire of mutational signatures in human cancer. Nature.

[44] van Griethuysen JJM (2017). Computational radiomics system to decode the radiographic phenotype. Cancer Res..

[45] Thibault G (2013). Shape and texture indexes application to cell nuclei classification. Int. J. Pattern Recognit. Artif. Intell..

[46] Amadasun M, King R (1989). Textural features corresponding to textural properties. IEEE Trans. Syst. Man. Cybern..

[47] Galloway MM (1975). Texture analysis using gray level run lengths. Comput. Graph. Image Process..

[48] Sun C, Wee WG (1983). Neighboring gray level dependence matrix for texture classification. Comput. Vis. Graph. Image Process..

[49] Haralick RM, Shanmugam K, Dinstein I (1973). Textural features for image classification. IEEE Trans. Syst. Man Cybern..

[50] Hanna MG (2019). Whole slide imaging equivalency and efficiency study: experience at a large academic center. Mod. Pathol..

[51] van der Walt S (2014). scikit-image: image processing in Python. PeerJ.

[52] Schmidt, U., Weigert, M., Broaddus, C. & Myers, G. Cell detection with star-convex polygons. In *Medical Image Computing and Computer Assisted Intervention (MICCAI)* 265–273 (Springer International Publishing, 2018).

[53] Bankhead P (2017). QuPath: Open source software for digital pathology image analysis. Sci. Rep..

[54] Benjamini Y, Hochberg Y (1995). Controlling the false discovery rate: a practical and powerful approach to multiple testing. J. R. Stat. Soc. B Method..

[55] Davidson-Pilon C (2019). lifelines: survival analysis in Python. J. Open Source Softw..

