# Supplementary material for: Multimodal data integration using machine learning improves risk stratification of high-grade serous ovarian cancer
Source: Nat Cancer. 2022 Jun 28;3(6):723–33. doi: 10.1038/s43018-022-00388-9 (PMC9239907; doi:10.1038/s43018-022-00388-9)
Supplement: Supplementary file 1 — List of MSK MIND Consortium members. [file 43018_2022_388_MOESM1_ESM.pdf]

nature portfolio

https://doi.org/10.1038/s43018-022-00388-9

Supplementary information

Multimodal data integration using machine learning improves risk stratification of high-grade serous ovarian cancer

In the format provided by the authors and unedited

MSK MIND Consortium Members

Name	Department
Sohrab P. Shah, PhD	Epidemiology & Biostatistics
Jianjiong Gao, PhD	Epidemiology & Biostatistics
Paul Sabbatini, MD	Medicine
Peter D. Stetson, MD	Medicine
Nathaniel Swinburne, MD	Radiology
Nikolaus Schultz, PhD	Epidemiology & Biostatistics
Matthew Hellmann, MD	Medicine
Yulia Lakhman, MD	Radiology
Mithat Gonen, PhD	Epidemiology & Biostatistics
Pedram Razavi, MD, PhD	Medicine
Elizabeth Sutton, MD	Radiology
Pegah Khosravi, PhD	Epidemiology & Biostatistics
Kevin Boehm, PhD	Graduate Medical Education
Rami Vanguri, PhD	Epidemiology & Biostatistics
Justin Jee, MD, PhD	Medicine
Karl Pichotta, PhD	Epidemiology & Biostatistics
Christopher Fong, PhD	Epidemiology & Biostatistics
Arfath Pasha	Epidemiology & Biostatistics
Doori Rose	Epidemiology & Biostatistics
Essam Elsherif	Epidemiology & Biostatistics
Andrew Aukerman	Epidemiology & Biostatistics
Druv Patel	Epidemiology & Biostatistics
Anika Begum	Epidemiology & Biostatistics
Elizabeth Zakszewski, PhD	Epidemiology & Biostatistics
Benjamin Gross	Center for Molecular Oncology
John Philip	Health Informatics
Luke Geneslaw	Pathology
Robert Pimienta	Digital Informatics and Technology Solutions
Surya Narayana Rangavajhala	Digital Informatics and Technology Solutions
