# Supplementary material for: Multimodal data integration using machine learning improves risk stratification of high-grade serous ovarian cancer
Source: Nat Cancer. 2022 Jun 28;3(6):723–33. doi: 10.1038/s43018-022-00388-9 (PMC9239907; doi:10.1038/s43018-022-00388-9)
Supplement: Supplementary file 2 — Reporting Summary [file 43018_2022_388_MOESM2_ESM.pdf]

nature portfolio

Corresponding author(s): Sohrab P Shah, PhD

Last updated by author(s): 2022/04/04

# Reporting Summary

Nature Portfolio wishes to improve the reproducibility of the work that we publish. This form provides structure for consistency and transparency in reporting. For further information on Nature Portfolio policies, see our Editorial Policies and the Editorial Policy Checklist.

## Statistics

For all statistical analyses, confirm that the following items are present in the figure legend, table legend, main text, or Methods section.

| n/a | Confirmed | |
|---|---|---|
| ☐ | ☒ | The exact sample size ($n$) for each experimental group/condition, given as a discrete number and unit of measurement |
| ☒ | ☐ | A statement on whether measurements were taken from distinct samples or whether the same sample was measured repeatedly |
| ☐ | ☒ | The statistical test(s) used AND whether they are one- or two-sided<br>*Only common tests should be described solely by name; describe more complex techniques in the Methods section.* |
| ☐ | ☒ | A description of all covariates tested |
| ☐ | ☒ | A description of any assumptions or corrections, such as tests of normality and adjustment for multiple comparisons |
| ☐ | ☒ | A full description of the statistical parameters including central tendency (e.g. means) or other basic estimates (e.g. regression coefficient) AND variation (e.g. standard deviation) or associated estimates of uncertainty (e.g. confidence intervals) |
| ☐ | ☒ | For null hypothesis testing, the test statistic (e.g. $F$, $t$, $r$) with confidence intervals, effect sizes, degrees of freedom and $P$ value noted<br>*Give P values as exact values whenever suitable.* |
| ☒ | ☐ | For Bayesian analysis, information on the choice of priors and Markov chain Monte Carlo settings |
| ☒ | ☐ | For hierarchical and complex designs, identification of the appropriate level for tests and full reporting of outcomes |
| ☐ | ☒ | Estimates of effect sizes (e.g. Cohen's $d$, Pearson's $r$), indicating how they were calculated |

*Our web collection on statistics for biologists contains articles on many of the points above.*

## Software and code

Policy information about availability of computer code

| | |
|---|---|
| Data collection | The electronic healthcare record  was used to acquire clinical outcomes, and PACS (GE Centricity v 7.0) was used to review CT scans for quality. H&E WSIs were reviewed and segmented in the institutional slide viewer web application. cBioPortal was used to acquire genomic information. For the TCGA, the TCIA was used to acquire CT images, the GDC Portal was used to acquire H&E WSIs, and cBioPortal was used to acquire genomic information. |
| Data analysis | Analysis was conducted in QuPath 0.2.3 (with the StarDist extension), ITK SNAP 3.8.0, and  custom code written in Python 3.9.4 (using Pandas 1.2.4, NumPy 1.20.2, PyTorch 1.5.1, TorchVision 0.6, OpenSlide 1.1.1, Seaborn 0.11.1, Matplotlib 3.4.2, SciPy 1.6.3, scikit-learn 0.24.0, PyRadiomics 3.0, and Lifelines 0.25.7. Conda environments are provided to streamline reproduction. |

For manuscripts utilizing custom algorithms or software that are central to the research but not yet described in published literature, software must be made available to editors and reviewers. We strongly encourage code deposition in a community repository (e.g. GitHub). See the Nature Portfolio guidelines for submitting code & software for further information.

## Data

Policy information about availability of data

All manuscripts must include a data availability statement. This statement should provide the following information, where applicable:

- Accession codes, unique identifiers, or web links for publicly available datasets
- A description of any restrictions on data availability
- For clinical datasets or third party data, please ensure that the statement adheres to our policy

DNA sequencing, H&E WSI, and CT data that support the findings of this study have been deposited at Synapse (Sage Bionetworks) under the accession code syn25946117. Additional H&E WSI, CT imaging, and genomic data were derived from the TCGA Research Network: http://cancergenome.nih.gov/ and The Cancer

Imaging Archive: https://www.cancerimagingarchive.net/. Raw data from MSK-IMPACT performed in the CLIA lab in the Department of Pathology is not currently permitted in public repositories because ethical and legal implications are still being discussed at an institutional level: thus, the derivative features related to HRD status are shared in the repository. Source data have been provided as Source Data files. All other data supporting the findings of this study are available from the corresponding author on reasonable request.

# Field-specific reporting

Please select the one below that is the best fit for your research. If you are not sure, read the appropriate sections before making your selection.

☒ Life sciences ☐ Behavioural & social sciences ☐ Ecological, evolutionary & environmental sciences

For a reference copy of the document with all sections, see nature.com/documents/nr-reporting-summary-flat.pdf

# Life sciences study design

All studies must disclose on these points even when the disclosure is negative.

| | |
|---|---|
| Sample size | No statistical method was used to predetermine sample size. After reviewing patients with biopsy-proven HGSOC and at least one of the following two conditions: (1) CE-CT available in PACS at our institution or (2) H&E WSI digitized at our institution or source tissue available before chemotherapy, we assembled a dataset of 444 patients. Data were excluded from the analyses only for the reasons detailed in the methods and prior to any machine learning modeling. |
| Data exclusions | Exclusion criteria were pre-established and included conflicting evidence for HRD status (without high-confidence higher-order feature such as signature 3 detected by SigMA), no tumor, excessive chatter, or intraparenchymal (non-soft tissue) lesions on H&E WSIs, and artifacts, low signal-to-noise ratio, or poor intravenous contrast bolus timing on CT. Every effort was made to include H&E WSIs with minimal tumor and CT images with imperfect quality to test potential clinical feasibility. |
| Replication | A test set were used to control for overfitting. Our cohort comprised both internal (MSKCC) and external H&E and CT images. Given the data science nature of the project, true replication (i.e., generating new data and testing the same relationships) will require further test set curation. Four-fold cross-validation was used to ensure generalizable performance of the H&E tissue type classifier. |
| Randomization | The test set was sampled randomly from patients with available CT imaging, H&E imaging, and HRD status. |
| Blinding | The investigators were not blinded to allocation during outcome assessment. Radiologists and pathologists did not have outcomes readily available during data annotation. |

# Reporting for specific materials, systems and methods

We require information from authors about some types of materials, experimental systems and methods used in many studies. Here, indicate whether each material, system or method listed is relevant to your study. If you are not sure if a list item applies to your research, read the appropriate section before selecting a response.

## Materials & experimental systems

| n/a | Involved in the study | |
|---|---|---|
| ☒ | ☐ | Antibodies |
| ☒ | ☐ | Eukaryotic cell lines |
| ☒ | ☐ | Palaeontology and archaeology |
| ☒ | ☐ | Animals and other organisms |
| ☐ | ☒ | Human research participants |
| ☒ | ☐ | Clinical data |
| ☒ | ☐ | Dual use research of concern |

## Methods

| n/a | Involved in the study | |
|---|---|---|
| ☒ | ☐ | ChIP-seq |
| ☒ | ☐ | Flow cytometry |
| ☒ | ☐ | MRI-based neuroimaging |

## Human research participants

Policy information about studies involving human research participants

| | |
|---|---|
| Population characteristics | The median age was 63 years [IQR 55-71] for the training set and 66 years [IQR 59-70] for the test set. All patients were female. All patients had biopsy-proven high-grade serous ovarian cancer. In the training set, 175 received neoadjuvant chemotherapy, 82 underwent primary debulking surgery, and 147 had unknown treatment. In the test set, 31 received neoadjuvant chemotherapy, 8 underwent primary debulking surgery, and 1 had unknown treatment. The training set contained 218 homologous recombination proficient and 119 homologous recombination deficient cases, and the test set contained 12 homologous recombination deficient and 28 homologous recombination proficient cases. |
| Recruitment | Participants were not recruited, but rather retrospectively identified from the institutional data warehouse as per the outlined criteria for data availability and biopsy-proven HGSOC diagnosis. |

| | |
|---|---|
| Ethics oversight | Memorial Sloan Kettering Cancer Center’s Institutional Review Board |

Note that full information on the approval of the study protocol must also be provided in the manuscript.
